# Annotating sonography-guided fine-needle aspiration cytology in the management of thyroid nodules in thyroidology

**DOI:** 10.1590/1806-9282.20250425

**Published:** 2025-07-07

**Authors:** Ilker Sengul, Demet Sengul

**Affiliations:** 1 Giresun University Faculty of Medicine, Division of Endocrine Surgery – Giresun, Turkey.; 2 Giresun University Faculty of Medicine, Department of General Surgery – Giresun, Turkey.; 3 Giresun University Faculty of Medicine, Department of Pathology – Giresun, Turkey.

Dear Editor,

Tyroidology, a dynamic discipline, deals with disorders and therapeutic options of the delicate papillon gland, *the thyroid*, which may demand a gracious approach in human beings^1,2^. We read with great interest and respect to the research article entitled "The Impact of Ultrasound-Guided Fine-Needle Aspiration Cytology in the Management of Thyroid Nodule." This beneficial research seems to demand a determining efficacy of the ultrasonography (US)-guided fine-needle aspiration (FNA) in thyroid nodules by comparing palpation-guided FNA in 456 outpatients over 12 months^3^. Riju et al.^3^ declared in their study that US-FNA utilization attenuated the inadequacy. The authors remarked in their work that they used 24-gauge (G) needles for the interventional procedures of the thyroid nodules. They emphasized not using any local anesthetic agents for these sampling procedures. Riju et al.^3^ pointed out that the Bethesda System for Reporting Thyroid Cytopathology (TBSRTC), 3rd edition (ed), was used to evaluate the cytology of suspicious thyroid nodules. However, a wide range of needle sizes, 20–27-G, have been used for FNA applications in different geographic regions worldwide, that is, 25–27-G in most Western countries and 21–22-G in Japan^4-8^. In addition, we mentioned Category III, TBSRTC, 3rd ed., from another perspective and recommended the requirement of zooming in thyroid nodules in suspense, 10–15 mm with repeat cytology in the 2nd issue, Volume 67, *Revista da Associação Médica Brasileira* ^9^. Moreover, we emphasized whether it is essential to maintain Category III, TBSRTC, 3rd ed., as a unique and indivisible category within indeterminate cytology of thyroid nodules or not, published in the 10th issue, Volume 67, *Revista da Associação Médica Brasileira* ^10^, prior to the 3rd edition of this lexicon, the 2023 TBSRTC, by Ali et al.^11^ in *Thyroid*, which has been announced after two former successful editions by Cibas et al.^12,13^ Lastly, we have recommended working with subsets to resolve the ongoing debate on "indeterminate cytology," similar to "intermediate suspicion" in radiology^14^. Therefore, would the utilization of thicker or finer needles alter^1,2,4-8^ the outcome(s) of this study? Furthermore, would harnessing a topical and/or local anesthesia prior to the procedure transmute^1,2,4,5,7^ the desinence(s) of this work? Moreover, would a feasible application of the novel subgroups of Category III, TBSRTC, 3rd ed, switch or modify the consequence(s) of this study in thyroidology?^9,11-15^ This issue merits further investigation. We thank Riju et al.^3^ for their valuable study.

## Data Availability

The datasets generated and/or analyzed during the current study are available from the corresponding author upon reasonable request.
